# Analysis of Protein–Protein Interactions in MCF-7 and MDA-MB-231 Cell Lines Using Phthalic Acid Chemical Probes

**DOI:** 10.3390/ijms151120770

**Published:** 2014-11-13

**Authors:** Shih-Shin Liang, Tsu-Nai Wang, Eing-Mei Tsai

**Affiliations:** 1 Department of Biotechnology, College of Life Science, Kaohsiung Medical University, Kaohsiung 80708, Taiwan; 2 Department of Public Health, College of Health Science, Kaohsiung Medical University, Kaohsiung 80708, Taiwan; E-Mail: wangtn@kmu.edu.tw; 3 Graduate Institute of Medicine, College of Medicine, Kaohsiung Medical University, Kaohsiung 80708, Taiwan; 4 Center for Resources, Research and Development, Kaohsiung Medical University, Kaohsiung 80708, Taiwan; 5 Center of Excellence for Environmental Medicine, Kaohsiung Medical University, Kaohsiung 80708, Taiwan

**Keywords:** phthalate, phthalic acid, protein–protein interaction, MCF-7, MDA-MB-231

## Abstract

Phthalates are a class of plasticizers that have been characterized as endocrine disrupters, and are associated with genital diseases, cardiotoxicity, hepatotoxicity, and nephrotoxicity in the GeneOntology gene/protein database. In this study, we synthesized phthalic acid chemical probes and demonstrated differing protein–protein interactions between MCF-7 cells and MDA-MB-231 breast cancer cell lines. Phthalic acid chemical probes were synthesized using silicon dioxide particle carriers, which were modified using the silanized linker 3-aminopropyl triethoxyslane (APTES). Incubation with cell lysates from breast cancer cell lines revealed interactions between phthalic acid and cellular proteins in MCF-7 and MDA-MB-231 cells. Subsequent proteomics analyses indicated 22 phthalic acid-binding proteins in both cell types, including heat shock cognate 71-kDa protein, ATP synthase subunit beta, and heat shock protein HSP 90-beta. In addition, 21 MCF-7-specific and 32 MDA-MB-231 specific phthalic acid-binding proteins were identified, including related proteasome proteins, heat shock 70-kDa protein, and NADPH dehydrogenase and ribosomal correlated proteins, ras-related proteins, and members of the heat shock protein family, respectively.

## 1. Introduction

Phthalates such as di-(2-ethylhexyl) phthalate (DEHP), butyl benzyl phthalate (BBP), di-isononyl phthalate (DINP), di-isodecyl phthalate (DIDP), di-ethyl phthalate (DEP), di-isobutyl phthalate (DIBP), and di-n-butyl phthalate (DBP) are globally used to increase the plasticity of plastic products, and are easily detected in bags, infants’ toys, bottles, flooring, and cosmetics. Moreover, chronic kidney disease (CKD) patients undergoing hemodialysis are exposed to DEHP and polyvinyl chloride (PVC) from dialysis tubes and bag filters [1,2]. The phthalates DEHP, BBP, and DBP and their metabolites are classified as endocrine disruptors, and have been shown to adversely affect sexual development in rats [3,4,5] and male rabbits exposed to DBP during adolescence [6]. Moreover, an increased concentration of phthalates’ metabolites reportedly induced asthma and allergic diseases, and they were easily detected in urine [7,8]. Accordingly, simulations of the Comparative Toxicogenomics Database (CTD) associate phthalates with cardiotoxicity, hepatotoxicity, and endocrine and genital diseases. In particular, the relationship between DEHP and mono-2-ethylhexyl phthalate MEHP and nephrotoxicity has been shown in GeneOntology pathways using network analyses with Ingenuity Pathways Analysis (IPA, QIAGEN’s Ingenuity Systems, Redwood, CA, USA), Pathway Studio (Ariadne, Inc., Rockville, MD, USA), and MetaCore Analytical Suite (GeneGO, Inc., St. Joseph, MI, USA) software [9]. The U.S. Centers for Disease Control and Prevention (CDC) announced that >97% of Americans had detectable phthalate metabolites in urine samples, including monobutyl phthalate (MBP), monobenzyl phthalate (MBzP), and monoethyl phthalate (MEP). In addition, urine samples from >75% of Americans contain DEHP and dimethyl phthalate (DMP) metabolites, such as MEHP and monomethyl phthalate (MMP) [10,11], and urine samples from >92% of Americans contain bisphenol A (BPA) [11,12].

Proteomic techniques using tandem mass spectrometry (MS) and bioinformatics have been developed rapidly over the past two decades. Whereas proteomics studies previously relied on gel electrophoresis [13], gel-free shotgun proteomics techniques are now widely used to screen target proteins [14]. However, tandem MS remains critical to the evaluation and verification of biomarkers after identification with genomics and quantitative proteomic comparison of normal and abnormal specimens [15,16]. Multidimensional gel electrophoresis or multidimensional liquid chromatography (MDLC) techniques can limit numbers of candidate biomarkers [17,18,19,20]. However, numbers of identified nonspecific biomarker candidates often hamper evaluation and verification. As alternatives, target protein screening can be achieved using activity-based chemical probes that detect proteomic profiles according to carbon electrophiles [21] or activity-based proteomics that generate serine hydrolase enzymes [22]. Similar methods involve the design of probes using click chemistry to connect proteins and carriers [21,23], the use of chemiluminescent bioprobes [24], and Au nanoparticles linked with synthetic DNA to detect estrogen receptors [25].

In previous studies, phthalic acid was observed as a secondary metabolite from phthalate derivation, which is observed in dialysis patients [2]. In addition, the phthalic acid metabolites of DEHP and MEHP were described [26]. In the present study, we used phthalic acid as a phthalate precursor to synthesize esterified phthalic acid chemical probes and detect protein–protein interactions. Previously, we developed chemical probes that generate phthalic acid or nicotinic acid using 3-aminopropyl triethoxyslane (APTES) linkers on silicon dioxide particles [27,28]. In our previous study, BBP promoted progression of a breast cancer cell line by inducing lymphoid enhancer factor 1 [29]. For the present study, we used chemical probes to characterize phthalic acid-binding proteins in MCF-7 and MDA-MB-231 cells. Subsequently, quantitative proteomics analyses identified 22 binding proteins that were common to both cell types, including heat shock cognate 71-kDa protein, ATP synthase subunit beta, and heat shock protein HSP 90-beta. Finally, ATP synthase subunit beta, heat shock protein HSP 90-beta, and heat shock cognate 71-kDa protein-linked proteasome protein were identified as exclusive MCF-7 proteins, and connected ribosomal correlated proteins were identified as specific to MDA-MB-231 cells.

## 2. Results and Discussion

### 2.1. Phthalic Acid Chemical Probe Synthesis and Characterization

Phthalic acid chemical probes were synthesized and characterized as shown in previous studies [27,28]. SiO_2_ surfaces were modified by APTES using a silanized modification technique [30]. After reaction of carboxylic groups with 1-Ethyl-3-(3-dimethylaminopropyl) carbodiimide (EDC)/*N-*hydroxysuccinimide (NHS), phthalic acid was generated for 12 h and synthetic probes were characterized using infrared spectroscopy (IR) [31].

### 2.2. Identification and Quantitation of Phthalic Acid-Binding Proteins Using Proteomics

**Figure 1:**
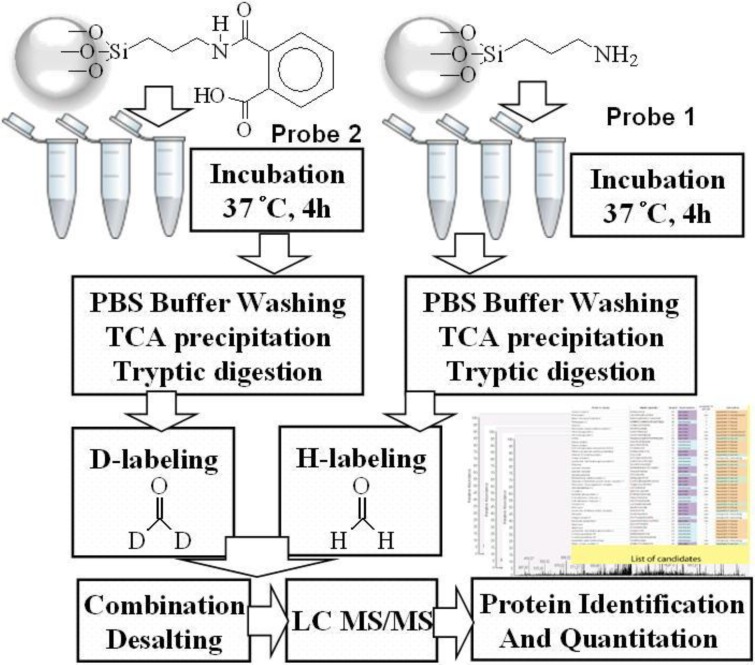
Flowchart of cell treatments and quantitation of proteins in MCF-7 and MDA-MB-231 cell using a phthalic acid chemical probe.

Chemical probes were individually incubated with MCF-7 and MDA-MB-231 cell lysates, and phthalic acid-bound proteins were identified using LC-MS/MS (Figure 1). After reduction by dl-dithiothreitol (DTT) and alkylation by iodoacetamide (IAM), related proteins were extracted and eluted using 1% sodium dodecyl sulfate (SDS). SDS was then removed by trichloroacetic acid (TCA) precipitation and proteins were subjected to tryptic digestion. Tryptic peptides bound to probes 1 and 2 were labeled with formaldehyde-*H_2_* and formaldehyde-*D_2_*, respectively. Labeled samples were acidized using 10% trifluoroacetic acid (TFA) and were then desalted using a C18 desalting cartridge. Subsequently, eluted samples containing peptide mixtures were examined using LC-MS/MS, and raw data were generated using Raw2MSM (version 1.10_2007.06.14) [32] for protein characterization and Mascot Distiller (version 2.4.2.0 (64 bits)) for protein quantitation.

### 2.3. Phthalic Acid-Binding Proteins in MCF-7 and MDA-MB-231 Cell Lines

Protein quantitation using Mascot Distiller and manual statistics showed that ATP synthase subunit beta, the heat shock protein family, and elongation factor 1-alpha 1 bound phthalic acid chemical probes, and related proteins were found in MCF-7 and MDA-MB-231 cell lines. Furthermore, phthalic acid chemical probes were bound to numerous proteasome-related and energy-correlated proteins such as NAD(P)H dehydrogenase, UDP-glucose 6-dehydrogenase, and fatty acid synthase in MCF-7 cells. Among the correlated proteins (Table 1), the phthalic acid probes detected ribosomal proteins such as 60S acidic ribosomal proteins and 40S ribosomal proteins, ras-related proteins, and heat shock proteins including 60-kDa heat shock protein and heat shock 70-kDa protein 6.

**Table 1:** Phthalic acid chemical probe related proteins with significant D/H labeling ratios in MCF-7 and MDA-MB-231 breast cancer cell lines.

Accession ^a^	Gene Name ^b^	Protein Identification	MDA-MB-231 H/L(I) ^c^	MDA-MB-231 H/L(II) ^c^	MDA-MB-231 H/L(III) ^c^	Average	MCF-7 H/L(I) ^c^	MCF-7 H/L(II) ^c^	MCF-7 H/L(III) ^c^	Average
*Phthalic acid-binding proteins identified in MCF-7 and MDA-MB-231*										
HSP7C_HUMAN	HSPA8	Heat shock cognate 71-kDa protein	5.0	4.0	3.4	4.2	n.a.	0.4	8.7	4.6
ATPB_HUMAN	ATP5B	ATP synthase subunit beta, mitochondrial	5.9	3.5	3.0	4.1	21.4	2.2	5.0	9.5
HS90B_HUMAN	HSP90AB1	Heat shock protein HSP 90-beta	8.5	10	8.6	9.1	17	n.a.	13	15.0
HS90A_HUMAN	HSP90AA1	Heat shock protein HSP 90-alpha	12.6	15.6	8.0	12.1	21.4	2.3	11	11.7
NPM_HUMAN	NPM1	Nucleophosmin	4.5	6.6	4.1	5.1	22	2.2	10	11.4
G3P_HUMAN	GAPDH	Glyceraldehyde-3-phosphate dehydrogenase	4.6	3.3	2.2	3.4	11	1.3	8.1	6.8
LDHA_HUMAN	LDHA	l-lactate dehydrogenase A chain	7.6	9.1	6.9	7.9	16	1.47	10	9.2
ENOA_HUMAN	ENO1	Alpha-enolase	4.4	3.2	5.0	4.2	13.8	2.9	9.2	8.6
KPYM_HUMAN	PKM2	Pyruvate kinase isozymes M1/M2	5.4	7.3	5.8	6.2	9.1	3.1	3.2	5.1
ADT2_HUMAN	SLC25A5	ADP/ATP translocase 2	7.1	5.2	8.1	6.8	47.1	3.1	12.0	20.7
ARF4_HUMAN	ARF4	ADP-ribosylation factor 4	7.0	6.8	14.1	9.3	30.3	n.a.	n.a.	30.3
TBA1B_HUMAN	TUBA1B	Tubulin alpha-1B chain	8.0	8.1	7.4	7.8	15.3	3.5	15.1	11.3
TBB2C_HUMAN	TUBB2C	Tubulin beta-2C chain	16.0	5.4	7.9	9.8	14.2	1.1	n.a.	7.7
TBB5_HUMAN	TUBB	Tubulin beta chain	12.1	5.1	8.2	8.5	14.1	1.0	0.9	5.3
RS2_HUMAN	RPS2	40S ribosomal protein S2	3.2	4.1	5.3	4.2	2.8	1.1	n.a.	2.0
PHB2_HUMAN	PHB2	Prohibitin-2	3.4	2.9	5.0	3.8	22.9	1.7	13.0	12.5
CATD_HUMAN	CTSD	Cathepsin D	3.3	2.5	2.5	2.8	8.2	2.0	6.5	5.6
K2C8_HUMAN	KRT8	Keratin, type II cytoskeletal 8	3.2	3.3	2.0	2.8	6.9	2.6	3.7	4.4
K1C18_HUMAN	KRT18	Keratin, type I cytoskeletal 18	3.5	2.4	3.1	3.0	18.5	n.a.	15.6	17.1
ACTB_HUMAN	ACTB	Actin, cytoplasmic 1	9.3	5.5	6.5	7.1	13.9	4.2	8.6	8.9
EF1A1_HUMAN	EEF1A1	Elongation factor 1-alpha 1	5.2	4.7	4.9	4.9	19.1	3.0	n.a.	11.1
TERA_HUMAN	VCP	Transitional endoplasmic reticulum ATPase	0.7	4.3	3.7	2.9	50.3	n.a.	n.a.	50.3
*Phthalic acid-binding proteins identified in MCF-7*										
PSA4_HUMAN	PSMA4	Proteasome subunit alpha type-4	-	-	-	-	6.4	0.8	5.1	4.1
PSB6_HUMAN	PSMB6	Proteasome subunit beta type-6	-	-	-	-	6.3	0.9	3.5	3.6
PSB5_HUMAN	PSMB5	Proteasome subunit beta type-5	-	-	-	-	6.0	0.7	3.0	3.2
PSA6_HUMAN	PSMA6	Proteasome subunit alpha type-6	-	-	-	-	5.9	0.8	4.2	3.6
PSA7_HUMAN	PSMA7	Proteasome subunit alpha type-7	-	-	-	-	5.8	0.8	4.7	3.8
PSA2_HUMAN	PSMA2	Proteasome subunit alpha type-2	-	-	-	-	5.3	1.0	4.6	3.6
PSA1_HUMAN	PSMA1	Proteasome subunit alpha type-1	-	-	-	-	5.3	0.9	4.6	3.6
PSB7_HUMAN	PSMB7	Proteasome subunit beta type-7	-	-	-	-	4.5	0.8	4.3	3.2
PSB2_HUMAN	PSMB2	Proteasome subunit beta type-2	-	-	-	-	8.0	n.a.	3.5	5.8
PSA3_HUMAN	PSMA3	Proteasome subunit alpha type-3	-	-	-	-	12.4	0.7	n.a.	6.6
PSB1_HUMAN	PSMB1	Proteasome subunit beta type-1	-	-	-	-	26.2	0.8	4.5	10.5
HSP71_HUMAN	HSPA1A	Heat shock 70-kDa protein 1A/1B	-	-	-	-	16.7	n.a.	n.a.	16.7
HS71L_HUMAN	HSPA1L	Heat shock 70-kDa protein 1-like	-	-	-	-	n.a.	2.1	7.5	4.8
NQO1_HUMAN	NQO1	NAD(P)H dehydrogenase [quinone] 1	-	-	-	-	33.4	2.9	n.a.	18.2
UGDH_HUMAN	UGDH	UDP-glucose 6-dehydrogenase	-	-	-	-	22.5	1.4	n.a.	12.0
ADT3_HUMAN	SLC25A6	ADP/ATP translocase 3	-	-	-	-	19.0	n.a.	n.a.	19.0
FAS_HUMAN	FASN	Fatty acid synthase	-	-	-	-	16.6	2.7	9.6	9.6
H2B1A_HUMAN	HIST1H2BA	Histone H2B type 1-A	-	-	-	-	16.9	2.3	10.4	9.9
K1C19_HUMAN	KRT19	Keratin, type I cytoskeletal 19	-	-	-	-	2.2	0.7	3.9	2.3
EF1A2_HUMAN	EEF1A2	Elongation factor 1-alpha 2	-	-	-	-	n.a.	3.0	10.1	6.6
VDAC1_HUMAN	VDAC1	Voltage-dependent anion-selective channel protein 1	-	-	-	-	6.6	1.7	6.1	4.8
*Phthalic acid-binding proteins identified in MDA-MB-231*										
RLA0_HUMAN	RPLP0	60S acidic ribosomal protein P0	10.4	n.a.	3.3	6.9	-	-	-	-
RL7A_HUMAN	RPL7A	60S ribosomal protein L7a	2.9	n.a.	n.a.	2.9	-	-	-	-
RL6_HUMAN	RPL6	60S ribosomal protein L6	6.5	4.6	2.7	4.6	-	-	-	-
RL23A_HUMAN	RPL23A	60S ribosomal protein L23a	2.5	2.9	5.1	3.5	-	-	-	-
RL5_HUMAN	RPL5	60S ribosomal protein L5	5.8	18.6	8.9	11.1	-	-	-	-
RL11_HUMAN	RPL11	60S ribosomal protein L11	3.9	n.a.	n.a.	3.9	-	-	-	-
RL31_HUMAN	RPL31	60S ribosomal protein L31	1.9	2.3	3.3	2.5	-	-	-	-
RS7_HUMAN	RPS7	40S ribosomal protein S7	2.4	2.4	5.8	3.5	-	-	-	-
RS13_HUMAN	RPS13	40S ribosomal protein S13	2.1	2.0	3.9	2.7	-	-	-	-
RS23_HUMAN	RPS23	40S ribosomal protein S23	3.2	3.9	4.7	3.9	-	-	-	-
PSMD2_HUMAN	PSMD2	26S proteasome nonATPase regulatory subunit 2	5.6	n.a.	33.7	19.7	-	-	-	-
CH60_HUMAN	HSPD1	60 kDa heat shock protein, mitochondrial	6.4	6.5	4.8	5.9	-	-	-	-
HSP76_HUMAN	HSPA6	Heat shock 70-kDa protein 6	3.9	n.a.	n.a.	3.9	-	-	-	-
RB11A_HUMAN	RAB11A	Ras-related protein Rab-11A	3.9	4.8	7.6	5.4	-	-	-	-
RAB10_HUMAN	RAB10	Ras-related protein Rab-10	n.a.	4.3	7.6	6.0	-	-	-	-
PTRF_HUMAN	PTRF	Polymerase I and transcript release factor	3.4	3.6	2.3	3.1	-	-	-	-
VIME_HUMAN	VIM	Vimentin	3.1	n.a.	1.7	2.4	-	-	-	-
ACTN1_HUMAN	ACTN1	Alpha-actinin-1	3.5	n.a.	n.a.	3.5	-	-	-	-
ACTN4_HUMAN	ACTN4	Alpha-actinin-4	n.a.	7.8	7.9	7.9	-	-	-	-
ATPA_HUMAN	ATP5A1	ATP synthase subunit alpha, mitochondrial	5.3	4.7	5.6	5.2	-	-	-	-
LDHB_HUMAN	LDHB	L-lactate dehydrogenase B chain	5.9	12.7	13.4	10.7	-	-	-	-
PSA5_HUMAN	PSMA5	Proteasome subunit alpha type-5	2.0	1.6	0.7	1.4	-	-	-	-
YBOX1_HUMAN	YBX1	Nuclease-sensitive element-binding protein 1	1.3	2.7	1.3	1.8	-	-	-	-
EF1G_HUMAN	EF1G	Elongation factor 1-gamma	6.8	0.7	13.8	7.1	-	-	-	-
RAN_HUMAN	RAN	GTP-binding nuclear protein Ran	5.8	6.6	5.8	6.1	-	-	-	-
H12_HUMAN	HIST1H1C	Histone H1.2	2.6	3.4	92.6	32.9	-	-	-	-
SMD3_HUMAN	SNRPD3	Small nuclear ribonucleoprotein Sm D3	65.3	n.a.	67.9	66.6	-	-	-	-
NP1L1_HUMAN	NAP1L1	Nucleosome assembly protein 1-like 1	8.4	9.6	5.9	8.0	-	-	-	-
IF4A1_HUMAN	EIF4A1	Eukaryotic initiation factor 4A-I	7.8	5.6	6.3	6.6	-	-	-	-
ARF1_HUMAN	ARF1	ADP-ribosylation factor 1	7.4	n.a.	n.a.	7.4	-	-	-	-
SET_HUMAN	SET	Protein SET	6.9	8.6	3.7	6.4	-	-	-	-
ENPL_HUMAN	HSP90B1	Endoplasmin	6.9	7.7	8.3	7.6	-	-	-	-

^a^ Accessions are presented according to Mascot software; ^b^ Gene names are listed according to the STRING software; ^c^ Ratio values are listed according to analyses using Mascot Distiller software.

### 2.4. Identification of Related Proteins Using Phthalic Acid Chemical Probes

**Figure 2:**
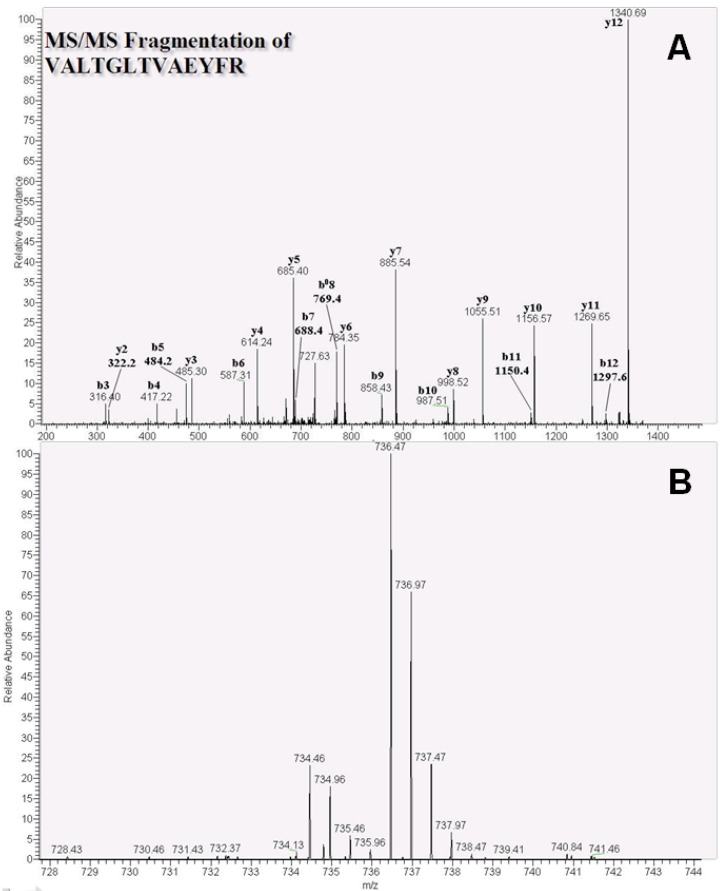
Nano-LC tandem MS spectra of the ATP synthase subunit beta protein peptide VALTGLTVAEYFR in MDA-MB-231 cells. (**A**) Product ion scan spectra of the identified peptide VALTGLTVAEYFR (*m*/*z* 736.47, 2^+^ charge); (**B**) The identified light (L, formaldehyde-*H_2_*) and heavy (H, formaldehyde-*D_2_*) isotope-labeled peptides (*m*/*z* 734.46 and 736.47, respectively).

Proteins were identified after conversion of raw MS data using Raw2MSM software with the Mascot search engine. ATP synthase subunit beta, heat shock protein HSP 90-beta, and heat shock cognate 71-kDa protein were characterized according to MS/MS patterns, and the peptide VALTGLTVAEYFR of the ATP synthase subunit beta protein showed b- and y-ion patterns (Figure 2A). Mean ATP synthase subunit beta protein quantities were 4.1- and 9.5-fold in MCF-7 and MDA-MB-231 cells (*n* = 3), respectively. Subsequent isotope labeling of VALTGLTVAEYFR using formaldehyde-*D_2_* and formaldehyde-*H_2_* (Figure 2B) gave an *m*/*z* of 734.46 with a charge 2^+^ and an *m*/*z* of 736.47, respectively.

**Figure 3:**
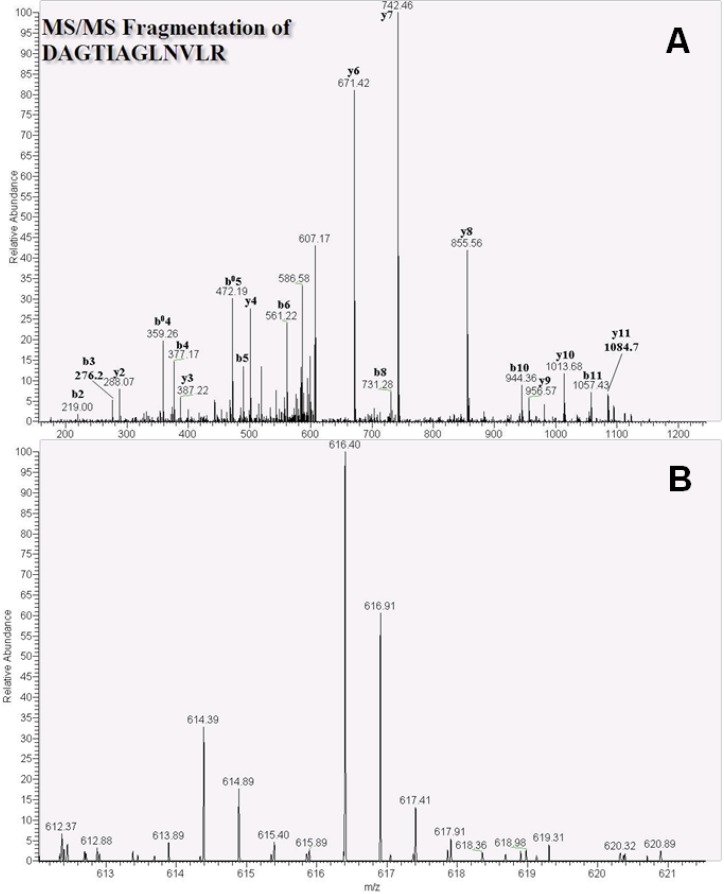
Nano-LC tandem MS analytic spectra of the heat shock cognate 71-kDa protein peptide DAGTIAGLNVLR in MDA-MB-231 cells. (**A**) Patterns of b- and y-ions were used to arrange the DAGTIAGLNVLR (*m*/*z* 616.40, 2^+^ charge) amino acid sequence; (**B**) Formaldehyde-*H_2_* and formaldehyde-*D_2_* isotope-labeled peptides with *m*/*z* 616.40 and 614.39, respectively.

**Figure 4:**
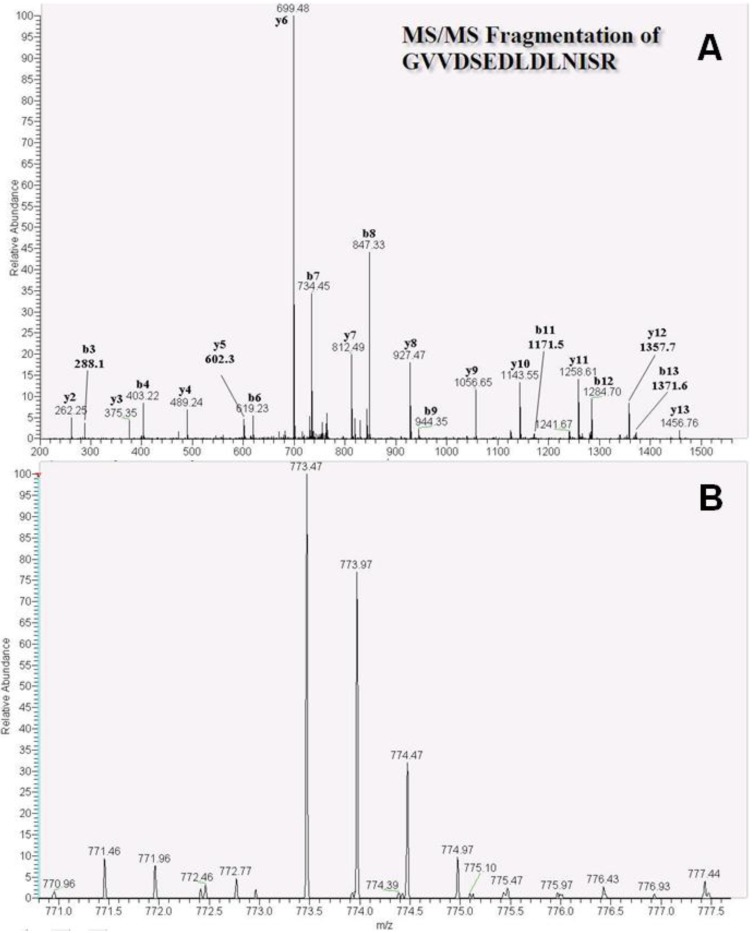
MS and MS/MS spectra of the heat shock protein HSP 90-beta peptide GVVDSEDLDLNISR. (**A**) MS/MS fragmentation of GVVDSEDLDLNISR (*m*/*z* 773.47, 2^+^ charge), showing b- and y-ion patterns; (**B**) Formaldehyde-*D_2_* labeled peptide (*m*/*z* 773.47) and formaldehyde-*H_2_* labeled peptide (*m*/*z* 771.46) were co-eluted using HPLC and had differing intensities for protein quantitation.

The heat shock cognate 71-kDa protein was also characterized using tandem MS, and the representative peptide DAGTIAGLNVLR was identified with a charge of 2^+^. MS/MS spectra (Figure 3A) and quantitative data showed 4.2- and 4.6-fold increases in this peptide in MCF-7 and MDA-MB-231 cells (*n* = 3), respectively with *m*/*z* values of 616.40 (D-labeled peptide) and 614.39 (H-labeled peptide; Figure 3B). Finally, the related heat shock protein HSP 90-beta (Figure 4A) and the peptide GVVDSEDLDLNISR were identified with a charge of 2^+^, along with a D-labeled peptide *m*/*z* of 773.47 and a H-labeled peptide *m*/*z* of 771.46 (Figure 4B). Finally, quantitative ratios showed 9.1- and 15.0-fold increases in MDA-MB-231 and MCF-7 cell lines, respectively.

### 2.5. Relationships between Protein–Protein Interactions in MCF-7 and MDA-MB-231 Cells

**Figure 5:**
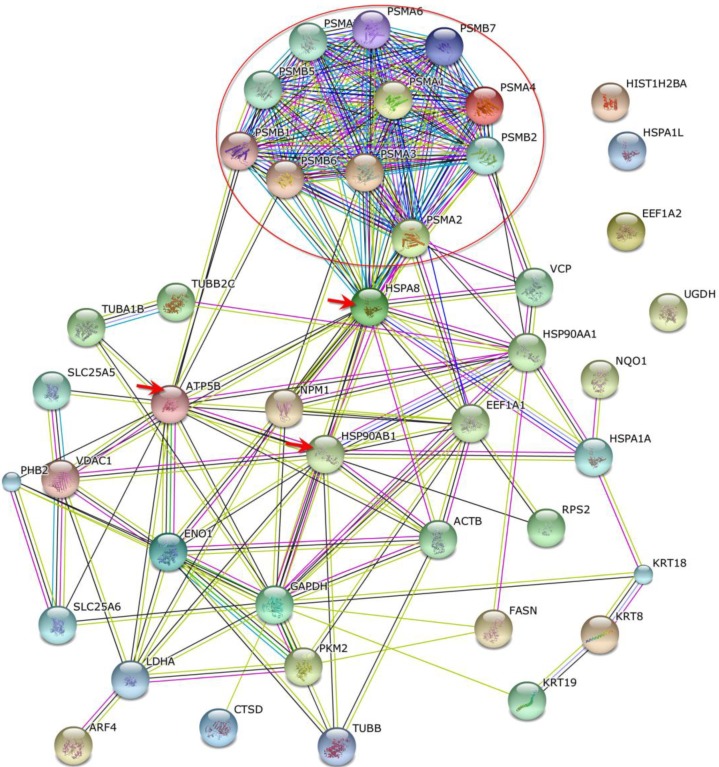
STRING protein–protein interactions between phthalic acid-binding proteins in MCF-7 cells. Red circles containing proteasome subunit proteins and the three red arrows indicate heat shock cognate 71-kDa protein, heat shock protein HSP 90-beta, and ATP synthase subunit beta.

**Figure 6:**
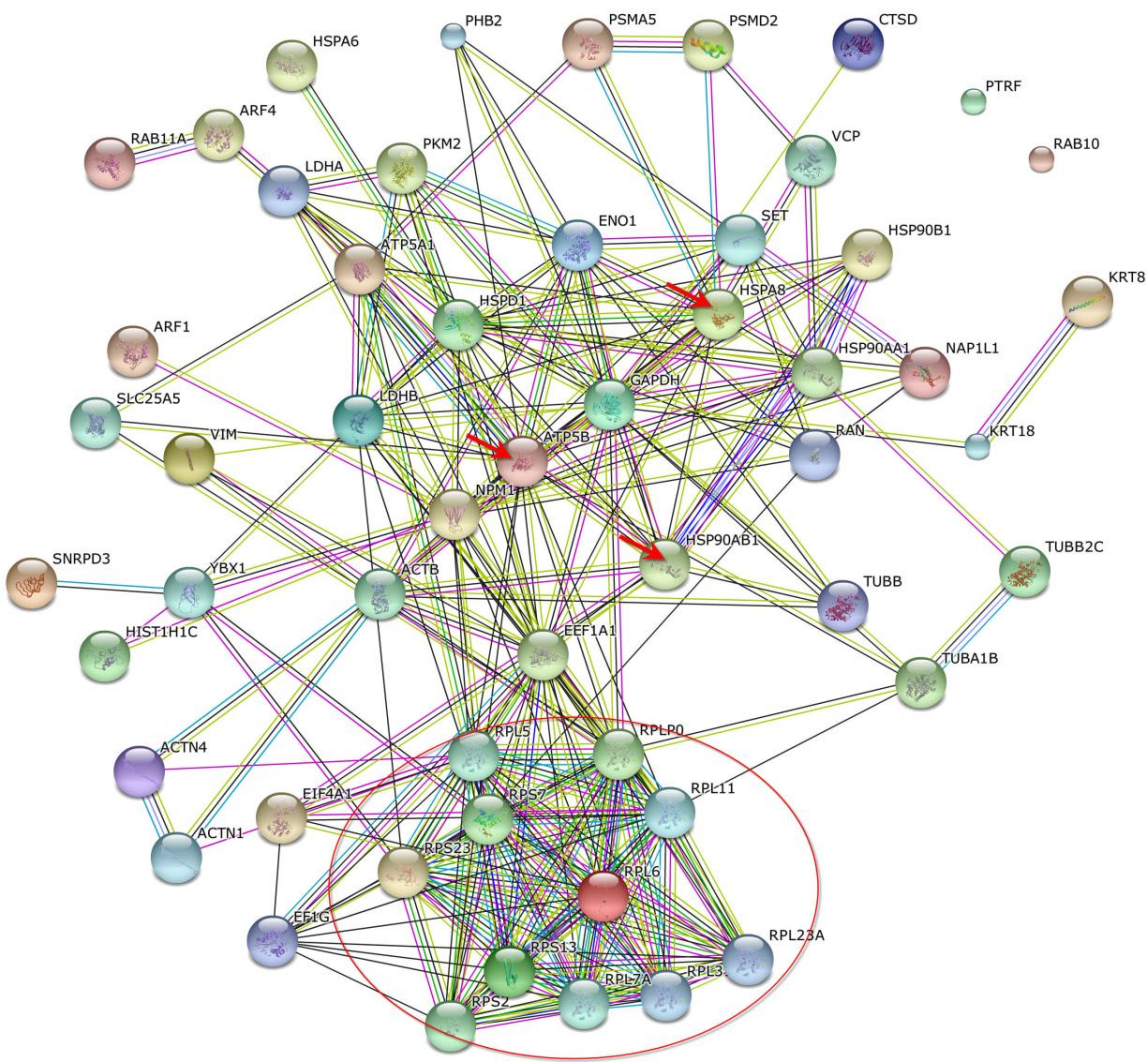
Schematic of the relationship between phthalic acid-binding proteins in MDA-MB-231 cells. High fold ratios were observed in triplicate experiments; the red circle includes numerous ribosomal proteins and the three red arrows indicate heat shock cognate 71-kDa protein, heat shock protein HSP 90-beta, and ATP synthase subunit beta.

Proteins that bind phthalic acid chemical probes were identified and relationships between these were characterized by organized protein–protein interactions using STRING software. Phthalic probes demonstrated arrangements of proteasome subunit proteins (red circle), and interactions with ATP synthase subunit beta (ATP5B) and heat shock 70-kDa protein 1A/1B (HSPA8) in MCF-7 cells (Figure 5). Furthermore, HSPA8 proteins interacted with heat shock protein HSP 90-beta (HSP90AB1), heat shock protein HSP 90-alpha (HSP90AA1), and nucleophosmin protein (NPM1). In MDA-MB-231 cells, protein–protein interactions (Figure 6) were found between HSPA8 and HSP90AB1, NPM1, ATP5B, glyceraldehyde-3-phosphate dehydrogenase (GAPDH), and elongation factor 1-alpha 1 (EEF1A1). Moreover, among related proteins, NPM1, GAPDH, HSP90 AB1, and ATP5B interacted with EEF1A1, which was correlated with a group of ribosomal proteins (red circle).

## 3. Experimental Section

### 3.1. Materials and Chemicals

dl-dithiothreitol (DTT), trifluoroacetic acid (TFA), 1-Ethyl-3-(3-dimethylaminopropyl) carbodiimide (EDC), sodium acetate, and sodium cyanoborohydride (NaBH_3_CN) were purchased from Sigma-Aldrich (St. Louis, MO, USA). Acetonitrile (MeCN), ammonium hydrogen carbonate (NH_4_HCO_3_), sodium hydroxide, hydrochloric acid, sodium dodecyl sulfate (SDS), acetic acid, ethanol, and urea were purchased from J.T.Baker (Phillipsburg, NJ, USA). Formaldehyde solution (36.5%–38% in H_2_O), phenylmethane sulfonyl fluoride (PMSF), leupeptin, sodium orthovanadate (Na_3_VO_4_), sodium chloride (NaCl), formic acid (FA), potassium chloride (KCl), potassium dihydrogen phosphate (KH_2_PO_4_), sodium dihydrogen phosphate (NaH_2_PO_4_), and iodoacetamide (IAM) were purchased from Sigma (St. Louis, MO, USA). Formaldehyde-*D_2_* (20% solution in D_2_O) was obtained from Isotec Corp. (Miamisburg, OH, USA). Trypsin was purchased from Promega (Madison, WI, USA). Phthalic acid, APTES, potassium bromide (FTIR grade), and *N-*hydroxysuccinimide (NHS) were purchased from Alfa Aesar (Heysham, UK). Deionized water was obtained with a resistance of 18.2 MΩ using a Millipore water system (Millipore, Bedford, MA, USA).

### 3.2. Synthesis and Characterization of Phthalic Acid Chemical Probes

The chemical probes were synthesized and characterized according to previous studies [27,28]. Briefly, 200 mg of silicon dioxide (SiO_2_, 400 mesh, approximately 40 μm; Acros Organics, Geel, Belgium) was activated using 0.5 M HCl and 0.5 M NaOH, then washed and dried with distilled water and ethanol to remove and evaporate HCl and NaOH. Surface silanization of SiO_2_ was performed by reacting with APTES (5% in ethanol), and the SiO_2_ was washed two times with 1 mL of ethanol and was baked overnight in an oven at 50 °C. Subsequently, 13 mg of EDC and 5 mg of NHS were added in 1 mL of deionized water to react with 10 mg of phthalic acid. After activation by EDC/NHS, the phthalic acid was conjugated to SiO_2_ via the amino groups of APTES. Functional groups of APTES-modified SiO_2_, phthalic acid SiO_2_, phthalic acid, and SiO_2_ were then identified using infrared spectroscopy (IR). Particles were ground to flat wafers with KBr (FTIR grade) under pressure, and were characterized using IR spectroscopy (Perkin-Elmer Spectrum RX1 spectrometer, Canton, MA, USA).

### 3.3. Culture of MCF-7 and MDA-MB-231 Breast Cancer Cells

MCF-7 and MDA-MB-231 cells were cultured in Dulbecco’s modified Eagle’s medium (DMEM, Sigma-Aldrich) supplemented with 1% penicillin (GibcoBRL, Grand Island, NY, USA) and 5% fetal bovine serum (FBS). Cells were cultured to 80% confluence in 100-mm dishes, at 37 °C in a 5% CO_2_ incubator. Cells were then lysed in a modified RIPA buffer containing 150 mM NaCl, 50 mM Tris-HCl, 1% NP-40, 0.1% SDS, 0.5 mM PMSF, 2 μg/mL leupeptin, and 1 mM Na_3_VO_4_ at pH 7.5. Protein concentrations of MCF-7 and MDA-MB-231 cell lysates were determined using the Bradford assay (Thermo, Rockford, IL, USA).

### 3.4. Chemical Probe Conditions for MCF-7 and MDA-MB-231 Cell Lysates

The individual and triplicate MCF-7 and MDA-MB-231 cell lysates containing 100 μg of protein were incubated with APTES-modified (10 mg; probe 1) and phthalic acid-modified (10 mg; probe 2) chemical probes diluted to 400 μL in a phosphate-buffer saline (PBS) containing 2.7 mM KCl, 137 mM NaCl, 8 mM NaH_2_PH_4_, and 1.4 mM KH_2_PO_4_, at 37 °C for 4 h. After centrifugation, supernatants were removed and chemical probes were washed with 200 μL of PBS buffer and incubated at 37 °C for 4 h, three times.

### 3.5. Tryptic Digestion and Quantitative Dimethyl Labeling

After centrifugation, supernatants were removed and protein-bound chemical probes were eluted in 0.1% SDS. Before tryptic digestion, SDS was removed by TCA precipitation and the extracted proteins were reduced by DTT, alkylated by IAM, and digested using 0.2 μg trypsin. After 4 h, an additional 0.2 μg of trypsin was added and samples were incubated at 37 °C for a further 18 h. Bound proteins on chemical probes were quantitated using dimethyl labeling, and tryptic peptide solutions were dried using a centrifuge vacuum [33]. Subsequently, lyophilized samples were redissolved in 180 μL of 100 mM sodium acetate at pH 5.5. Tryptic peptides bound to probe 1 were modified using 10 μL of formaldehyde-*H_2_*, and those bound to probe 2 were reacted with 10 μL of formaldehyde-*D_2_*. After 5 min of vortexing, labeled samples were reduced using 10 μL of 600 mM sodium cyanoborohydride for 1 h. Labeled solutions were then combined and desalted by adjusting the pH to 2–3 using 10% TFA/H_2_O. The desalting kit comprised a C18 reverse-phase chromatography column packed with C18 powder in an in-house column cartridge and holder. Eluted solutions were vacuum dried for nano-LC-MS/MS.

### 3.6. Nano-LC-Tandem MS Analysis, Protein Identification and Quantitation

The combined labeled and lyophilized fractions were redissolved in 10 μL of 0.1% FA in H_2_O and analyzed using a Thermo LTQ Orbitrap XL system (Thermo Fisher Scientific, San Jose, CA, USA). Separate analyses were performed using a Waters ACQUITY nanoflow system (nanoUPLC, Waters Corp., Manchester, UK). In these experiments, 3-μL aliquots of redissolved samples were injected into a C18 capillary pretrapped column (20 mm × 180 μm), and protein separation was performed using a reverse-phase Waters BEH C18 column (i.d. 75 μm × 150 mm, 1.7 μm particle size). UPLC flow rates were set at 5 μL/min (loading pump) and 300 nL/min (gradient pump); mobile phases were prepared in bottles A and B, which contained 0.1% FA in water and 0.1% FA in 100% MeCN, respectively. The linear gradient comprised 2% (B) for 2 min, 2%–40% (B) for 40 min, 40%–98% (B) for 8 min, 98% (B) for 2 min, 98% to approximately 2% (B) over 1 min, and then 2% (B) for 7 min. Separated peptides were nebulized using a voltage of 1.8 kV in the positive ion mode and detected by tandem MS using a scan mode of *m*/*z* 400–1600 Da with 30,000 resolutions in the Orbitrap chamber. Separated peptides were predominantly detected in the MS mode, and five with high-intensity signals were selected and transferred into a collision-induced dissociation (CID) chamber with nitrogen collision gas and 35 eV collision energy for MS/MS fragmentation. The second scan mode for the MS/MS analyzer was set at *m*/*z* 100–1600 Da, with exclusion of similar *m*/*z* ions, evasion of interferences, and a repeat duration of 30 s in data-dependent mode. The integrated UPLC loading and analytic pump was controlled by MassLynx 4.1 and Global ProteinLynx softwares, and MS data were managed and acquired using Xcalibur software (version 2.0.7, Thermo-Finnigan, Inc., San Jose, CA, USA). Raw MS data were converted to the Mascot generic data format by using Raw2MSM (version 1.10_2007.06.14) [32], and protein quantities were converted using Mascot Distiller software (version 2.4.2.0; 64 bits, Matrix Science Ltd., London, UK) with Orbitrap_res_MS2 (default parameter setting) for peak list transformation and Homo sapiens (human) taxonomy in the Swiss-Prot database for the Mascot search engine, coupled with the Universal Protein Resource (UniPort) 2013 database (accessed on 21 September 2011) [34]. The following parameter settings for the Mascot search program [35] were installed: allow up to zero missed cleavages for tryptic digestion, dimethylation [MD] for quantitation, carbamidomethyl cysteine set for fixed modifications, mass tolerance of 10 ppm with precursor ions, and 0.8 Da for fragment ions. Peptides that had charges of 1^+^, 2^+^, or 3^+^ and Mascot ion scores of >20 (*p* < 0.05, individual peptides) were selected. Subsequently, quantitative ratios of D-label (Probe 2)/H-label (Probe 1) were generated and listed for each protein.

### 3.7. Establishment of STRING Protein–Protein Interaction Networks

Interactions between proteins and phthalic acid were predicted using the STRING database (version 9.1), and relationships between phthalate-related proteins and associated proteins were demonstrated.

## 4. Conclusions

In this study, phthalic acid chemical probes were used to demonstrate relationships between phthalic acid-sensitive proteins in MCF-7 and MDA-MB-231 cells. In these experiments, phthalate-like (phthalic acid) structures interacted with a group of proteasome subunit proteins in MCF-7 cells, and with a group of ribosomal proteins in MDA-MB-231 cells. Moreover, these proteins were connected by heat shock cognate 71-kDa protein, ATP synthase subunit beta, and heat shock protein HSP 90-beta. Finally, protein networks in MCF-7 and MDA-MB-231 cells were established using STRING protein–protein interaction software. Future studies may identify phthalate receptors using chemical probes.
